# Supplementary material for: MicroRNAs Associated with Androgen Receptor and Metastasis in Triple-Negative Breast Cancer
Source: Cancers (Basel). 2024 Feb 4;16(3):665. doi: 10.3390/cancers16030665 (PMC10854913; doi:10.3390/cancers16030665)

Table S1. Original threshold cycle (CTs) values of miRNA PCR arrays for FFPE tissue samples												
	AR-positive, non-metastatic						AR-positive, metastatic					
	5695 ARP NM	70 ARP NM	802 ARP NM	847 ARP NM	1090 ARP NM	1919 ARP NM	2319 ARP M	7206 ARP M	7455 ARP M	8033 ARP M	1640 ARP M	2762 ARP M
**A1**	26.8	20.1	24.3	20.9	23	18.1	21.7	20.3	21.2	20.4	20.7	21.1
**A2**	25.4	21.6	24.3	21.4	26	19.7	21.4	19.3	20.1	19.9	22.2	22.4
**A3**	29.7	23.8	26.9	24.7	28.3	21.4	24.2	23.5	23.8	22.5	24.6	25.5
**A4**	30	24.9	27.3	23.9	28.3	21.9	24.7	24.3	24.1	23	24.8	26.7
**A5**	27.7	22.2	24.9	22.4	26.5	19.4	23.8	22.9	22.6	21.2	22.8	23.7
**A6**	28.9	23.1	24.5	22.1	25.9	20.4	26.8	24.8	25.1	24.5	22.9	23.1
**A7**	29.8	25.4	27.4	24.9	28	22.6	27.3	25.7	25.6	25.3	25.2	25.9
**A8**	28	23.8	26.2	23.4	27.4	20.8	25.4	23.9	23.8	25.4	24.1	23.6
**A9**	36.7	30.9	32.4	31.7	32.9	26.8	34.7	32.8	31.7	32.3	29.2	30.3
**A10**	29.3	22.2	23.8	22.7	23.1	19.1	25.1	23.8	24.1	23.7	21.8	21.9
**A11**	34.2	29.2	30.9	29.2	31.9	24.5	28.9	27.9	27.9	26.7	28.4	29.7
**A12**	32.6	26.2	27.5	25.3	28.6	23.9	30	28.2	28	28	25.4	24.6
**B1**	32.9	25.8	27.9	24.8	28.2	23.1	29.5	27.8	28.4	27.7	25	25
**B2**	27.9	20.2	22.7	21.3	22.8	18.2	21.8	20.5	21.7	20.2	19.9	20.6
**B3**	36.2	33.9	34.8	33.9	36	29.9	33.2	32.2	31.6	32.2	33.8	34
**B4**	33	29	30.1	26.1	31.2	25.1	28.9	27.2	27.8	27.7	26.8	28
**B5**	31.8	31.6	32.4	30.9	33	29.6	28.3	28.9	27.3	29.2	32.8	30.9
**B6**	30.7	24.7	27.7	25.8	28.7	23	25.5	25	25.7	24.8	25.1	25.7
**B7**	31.9	28	28.5	26	31	25.5	27.9	27.7	27.7	27.6	27	27.6
**B8**	30.8	27	29.8	27.6	30.7	25.9	28	26.4	26.7	27.2	28.3	29.6
**B9**	33.4	28.7	29.6	27.7	31	25.2	31.1	28.5	28	28.4	27.5	27.9
**B10**	27.1	26.9	26.9	23.4	25.5	23.7	24.8	23.9	24.7	23.2	24.9	23.8
**B11**	25.8	20.8	22.7	21.8	23	19.2	22.7	20.1	19.2	19.9	20.7	21.7
**B12**	28.9	24.4	22.9	21.9	25.1	21	25.9	23.8	23	24.4	23.4	22.3
**C1**	31.4	26.2	29.3	25.7	29.4	22.8	28.7	26	27.8	26.2	26	25.7
**C2**	31.4	27.5	28	26.8	29.4	25.5	25.6	26.9	26	27	28	27.8
**C3**	28.3	28.2	28.6	25.7	27.6	22.6	26.7	25.3	25.9	25.2	27.3	26.4
**C4**	28.5	25.3	26.7	23.7	27.1	21.9	24.7	23.9	24.6	24.2	25	25.1
**C5**	26.2	22.8	23.8	20.5	25.3	18.8	23	21.7	23.4	22.1	22	21.8
**C6**	29	26	27.8	23.7	28.5	22.9	27.1	26	26.4	25.2	23.7	24.7

**C7**	26.3	24.7	26.9	23.9	26.7	19.9	23.4	22.5	23.9	22.8	25.1	24.4
**C8**	27.4	25.8	27	24.4	28.7	20.8	23.9	23.5	23.7	23.9	26.3	25.5
**C9**	27.8	25.8	27.4	24.9	28.7	21	24.5	23.6	24.2	23.7	25.7	25.8
**C10**	29.8	29.5	30.9	27.3	32.3	25.5	26.7	26.3	26	26.1	29.5	29.4
**C11**	32.2	28.4	30.4	26.3	29.8	24.4	28.7	25.9	28	24.5	26.4	27.4
**C12**	29.3	27.1	27.1	24.7	28.6	23.6	25.5	26.2	24.4	25.5	26.3	26
**D1**	30.8	30	32.2	27.3	31.5	25.3	28.9	28.7	28.5	28.1	26.6	27.8
**D2**	27.7	24.8	25.6	22.9	26.2	22.9	24.4	21.9	22	22.9	23.7	22.8
**D3**	28.2	22.9	25.7	21.8	26.2	19.8	24.7	22.9	24	23.2	22.2	22.8
**D4**	27.4	22.3	24.7	21.8	25	18	24.7	22.1	24.2	22.8	22.2	23
**D5**	27.9	23.5	26.6	23.9	26.4	19.1	24.8	22.2	23.8	22.6	23.7	24
**D6**	29	25.7	27.5	23.2	27.7	22	27.7	26.3	26.3	26.4	23.5	24
**D7**	29	25.7	27.6	23.1	27	22.2	27.8	26.7	26.2	26.4	23.6	24.3
**D8**	29.5	27.2	27.7	23.8	27.9	23.8	27.3	25.9	27.7	26.4	26.2	26.2
**D9**	28	23.1	24.3	20.8	25.4	22	23	22.8	23.1	21.7	23	22.3
**D10**	25.9	22	22.6	20.3	23.2	21.2	20.8	20.3	20.8	19.1	22.6	20.4
**D11**	30.2	32.2	31.9	30.6	32.5	30.8	27.6	28.4	27	28.9	35	31.7
**D12**	34.7	28	30.3	24.9	29.8	26.5	26.9	29	26.2	28.4	27.2	26
**E1**	33.3	29	31.5	30.1	31.8	26.4	31.1	29.1	29.1	29.7	29.3	32.1
**E2**	27.2	21.2	25.3	26.5	24.8	20.7	21.7	21.5	21.3	21.5	21.4	21.9
**E3**	36.7	33.7	35	35.8	35.5	34.8	32.1	33.6	32.2	33.6	N/A	35.6
**E4**	27.9	24.7	26.8	23.1	27.8	21.8	26.1	25.1	25.3	24.5	22.6	23.5
**E5**	31	27.4	29.1	25	30.1	24.1	28.9	27.3	28.5	27.5	27.2	25.9
**E6**	25	19.9	22.2	18.6	21.7	17.3	22.6	20.4	20.4	20.4	18.5	18.4
**E7**	29.5	26	28.8	24.5	29.9	24.1	25.8	26.6	26.4	25.1	26.7	25.1
**E8**	30.4	28.8	29.7	24.2	29.5	27.2	29	28.3	27.9	27.4	28.6	29.7
**E9**	28.1	23.9	26.8	23.6	26.2	20.5	24.3	21.8	22.9	22.4	24.5	25.3
**E10**	27.9	24.7	25	22.8	26.2	21.1	24.8	23	23.2	22.8	23.8	23.9
**E11**	26.5	23.9	25.7	25.3	25.9	20.5	22.1	22.2	22.3	23	24	24.2
**E12**	32.3	26.2	25.9	24.8	27	23.2	26.1	26.5	25.1	26	24.3	26
**F1**	28.9	25.8	27.8	24.9	27.9	23.2	24.3	23.9	24.6	24	25.1	25.5
**F2**	27.1	20.9	22.4	21	23.3	19.6	23.5	21.1	22.2	21.1	20.8	21.9
**F3**	29.7	23.6	26.2	23.4	26.7	22.2	27.1	25	25.7	24.4	23.2	24.8
**F4**	27.2	23.8	25.4	22.8	25.7	20.4	24.5	22.9	22.8	22.9	22.7	22.5

**F5**	30.6	26.3	28.5	26.1	28.7	23.5	28.6	25.5	26.3	25.7	26	26.7
**F6**	28.4	24.3	25.4	23.5	26.2	21.7	23.8	22.7	23.3	22.8	23.3	24.2
**F7**	27.8	25.6	23.6	22.7	24.6	22.2	25.1	23.5	23.9	23.4	23.5	24.3
**F8**	26.6	23.7	23.9	22.6	25.9	21	23.6	22.7	22.4	23	22.6	23.1
**F9**	31.8	26.8	30.1	28.7	31.8	25.3	29.9	27.6	27.6	27.5	26.6	27
**F10**	27.3	27.5	27.8	27.4	25.9	26.8	25.5	24.7	24.4	25.1	28.2	28.8
**F11**	34.1	30.9	31.6	29.7	31.1	26.8	31.1	29.1	29.9	27.6	29.7	29.8
**F12**	29.2	24.9	28.7	24	27.7	21.4	27.3	24.9	24.7	23.9	24.5	26.2
**G1**	31.4	28.3	29.5	24.9	29.8	24.8	29.5	28.1	29.8	27.3	27	27
**G2**	35.9	31.9	35.8	32.6	35.5	30.9	31.7	31.4	31.4	31	33.2	32
**G3**	28.5	28	28.2	27.4	28.4	26.7	24.1	25	24.2	26	28.3	27.5
**G4**	33.7	29.8	33.2	29.7	32.9	28.1	32	30.1	29	30.5	30.1	30.4
**G5**	31	25.7	28.7	25.9	28.9	22.9	27.7	25.7	26.4	26	25.6	26.3
**G6**	34.2	34.7	35.8	33.7	36.7	33.5	31.9	32.4	30.9	32.8	34.7	34.5
**G7**	37	N/A	N/A	36.9	N/A	N/A	N/A	N/A	34.9	37.4	N/A	N/A
**G8**	33.4	33.7	33.5	29.7	33.6	32.1	32.5	32.5	29.9	31.9	34.8	34.3
**G9**	32.1	30.2	30	27.5	31.1	27.1	30.2	28.8	28.3	27.1	28.1	27.1
**G10**	29	26.4	27.6	24.8	28.8	22.6	25.6	24.6	25.9	24.2	24.3	25.1
**G11**	30	30.9	31.9	28.7	26.8	26.2	28	25.2	27.8	25	29.6	29
**G12**	32.8	28.3	29.6	26.4	30.5	24.6	30.5	29.5	28.1	27.6	27.1	27.5
**H01**	34.8	37.5	N/A	34.6	N/A	N/A	31.8	32.8	32.4	33.6	N/A	N/A
**H02**	36.1	N/A	N/A	36	36.7	37.3	32.5	33.8	33.1	34.4	N/A	N/A
**H03**	24.1	22.1	22.8	21.4	24.2	20.4	20	20	18.6	20	22.1	22.2
**H04**	23.7	22.2	22.2	22.1	22.9	20.9	20	19.9	18.2	19.7	22	22
**H05**	26.9	25.2	26	25.7	27.4	23.7	25.6	25.7	23.1	24.8	23.8	23.9
**H06**	24.1	21.6	21.8	21.2	23.2	19.8	19.3	18.9	17.7	19.7	22	22
**H07**	24.9	22.8	22.8	22.1	23.9	20.8	20.4	20.3	18.9	20.9	23.3	22.6
**H08**	23.8	21.5	21.2	20.4	22.9	21.3	23.1	21.4	20.3	21.7	20.5	20.5
**H09**	20.7	27	26.1	26.4	27.7	25.4	21.2	23	24	21.6	26.9	26.3
**H10**	21.4	27.9	27.3	27	27.8	27.1	22.9	22.8	24.7	21.3	26.6	26.6
**H11**	15.8	18.8	19.2	19.5	17.7	19.2	17.3	18.2	18.7	18.9	18.8	19
**H12**	18.9	19.7	19.5	19.7	18.1	19.6	19.4	19.4	19.4	19.1	18.9	19.2

Av	**24.6**	22.6	22.8	22.2	24.1	21.2	21.4	21.0	19.5	21.1	22.3	22.2
SD	1.2	1.4	1.7	1.9	1.7	1.3	2.4	2.4	2.0	2.0	1.2	1.1

AR-negative, non-metastatic						AR-negative, metastatic					
2036 ARN NM	2252 ARN NM	3382 ARN NM	5780 ARN NM	5994 ARN NM	7939 ARN NM	1863 ARN M	2285 ARN M	3736 ARN M	3930 ARN M	4570 ARN M	4910 ARN M
21.1	20	21.6	20.7	20.7	24	21	21.7	18.1	21.6	20.9	20.1
21.9	22	22.2	22.8	20.8	20.4	20.7	23.2	20.7	22.4	23.3	21.2
25.9	23.7	26.4	25.1	23.8	26.6	24.1	25.6	23.3	25.8	25.8	24.1
24.8	24.3	25	25.6	25.1	27.7	25.5	25.8	22.2	25.5	25.2	24.7
23.2	21.4	24.1	22.1	22.8	25.9	21.9	23.8	20.4	23.8	22.7	21.7
23.3	22.1	24.3	23	24.3	26.1	23.1	24.2	19.7	24.1	22.7	23.7
24.9	25.4	25.7	24.8	25.9	25.3	25.7	27	21.8	26.8	25.1	25.8
21.4	22.8	24.9	23.6	23.9	21.9	22.1	25	19.8	24.9	24.6	22.8
31.7	30.3	21.5	32.1	31.6	30	30.9	33	27.5	32.8	32.3	30
24.5	20.2	21.6	21.9	22.1	21.1	22	21.5	19.8	23.3	22.3	18.4
28.5	29.1	26.3	25.8	28.2	29.2	28.1	28.7	26.7	28.6	27.7	27.3
28.8	26.6	26.5	28.4	27.3	27	26.8	26.2	25	28.5	27.5	24.7
28.4	25.8	25.8	27.2	27.3	26.1	26.4	25.4	24.4	27.9	27	25.2
21.3	20.4	18.8	21.5	20.5	20.3	21.2	19.6	19	21.9	22.3	18.2
33.6	32.2	30.2	33.4	31.3	31.9	30.9	33.3	32.3	34.2	35.2	30.9
27.4	27.1	26.8	25.9	28.1	26.8	27.2	27.6	25	28.3	27	27.1
32.3	30.8	29.5	29.8	30.7	29.7	31.1	31.1	29.9	30.8	29.3	30.1
24.7	23.8	24.8	23	23.8	23.2	23.5	24.7	22.3	26.2	24.7	24
26.9	25.4	27	26.6	26.3	25	25.6	27.6	25.3	28.8	26.9	26.4
29.1	27.8	27.5	28.8	29.3	28.3	29.2	28.2	26	29.2	29	26.6
29.3	27.4	28.7	27.9	26.8	24.7	25.8	28.1	24.1	29	28.2	27.2
23.1	25.7	24.1	22.2	24.7	22.7	23	24.9	20.3	25.1	24.7	23.9
22.7	21.5	21.3	22.4	21.8	21.1	22.3	20.3	20.4	22.6	22.9	19.3
25.4	25	26.3	23.4	23.5	21.9	22	24.7	19.8	24.3	22.5	22.7
27.9	24.8	27.5	28.8	26	24.5	27	26.2	24.5	28.2	26.3	25
27.4	26.3	27.5	26.5	26.8	28.2	24.5	28.1	25.8	28.4	26.8	27.2
26.4	25.6	26.6	25.9	26.8	23.5	24.2	26.9	23.1	26.3	26.8	25.2
23.8	23.8	24.8	24	23.6	25.1	24.2	25.6	20.7	24.7	23.9	24.3
21.7	20.9	21.7	21.2	21.2	20.8	20.6	22.6	17.5	22.2	21	21.1
23	22.9	23.8	21.9	24.2	25.7	22.1	25.9	20.3	23.8	23.1	23.6

25.1	22.4	25.1	24.7	24.2	21.7	23.2	25	21.4	25.2	24.9	22.3
25	23.1	25.7	25.4	24.7	23.7	24	26.5	22.1	26.1	25.8	24.6
26	23.7	25.7	25.3	24.9	23.4	23.9	25.8	23.3	26.4	26	23.7
27.7	27.7	28.6	29	28	27.8	27.7	30.1	27.3	29.7	29	28.6
28.2	28.3	28.4	27.3	27.4	26.7	27.5	26.9	24.4	26.8	26.9	26.8
25.9	25.2	23.8	24.3	26.3	22.9	24.5	26.3	22.9	26.8	26.1	24.8
26.7	25.8	26.2	24.4	26.9	26.8	24.8	28.3	21.8	26.6	25.3	26.7
26.8	24.2	24.8	23.9	22.8	24.8	25	24.5	24	25.4	26.4	22.9
23.5	22.1	22.8	23.1	22.3	22.8	22.7	23.4	19.3	23.8	22.2	21.8
23.4	21.3	23.5	23.7	24.7	19.9	23.7	22.1	20.2	23.4	23.6	21.6
25	22.6	24	25.2	25.2	21.8	24.6	22.8	21.2	24.9	24.6	21.1
23.8	23	23	21.2	24.6	22.1	21.9	24	18.4	24.3	22.3	23.2
23.7	23.5	23	21.5	24.4	22.2	22.9	24.3	18.3	24.3	22.7	23
26.9	26.1	27.1	24.9	26.8	22.5	25.2	26.1	21	24.4	24.8	25.7
22.7	23.1	22.9	22.1	23.1	22	22	22.6	19.5	22.8	22.3	22.1
20.1	21.3	20.4	19.8	20.5	20.7	20.7	21.1	19.3	22.1	20.8	20.2
33.3	32.7	29.3	31.2	31.9	29.7	30.8	32.8	30.6	31.1	30.7	30.8
27.7	27.1	31.1	29	28	28.1	25.4	26	25.1	31.2	29.8	25.6
30	31.1	31.7	29.1	32.8	31.7	30.5	29.9	28.2	32.2	31.5	30.3
24.6	22.6	24.9	25.8	24	23.4	22.1	20.6	20.6	21.2	21.1	20.3
34.7	32.6	34	34.8	34.2	31.7	33.2	36.3	33.1	35.5	34.9	33.7
21.9	22	22.6	20.8	23	24.2	21.6	24.5	19.7	21.7	21.9	22.9
24.3	24.3	25.2	22.8	25.5	26.1	23.6	27.1	21.1	25.6	24.2	25.2
19.8	19.3	19.7	20.6	21.1	17.1	19.3	19.8	15.9	21	19	18.7
26.3	24.9	27.4	27.6	23.5	23.6	24	25.8	25.5	27.1	26.3	24.3
29.5	28.1	26.7	26.6	28.6	29.8	26.8	28.2	27.4	29.9	29.5	26.7
26.3	21.5	24.5	25.9	26	25.1	25.7	24.1	23.4	25.6	26	22
25.3	22.5	23.8	24	23.4	20.9	22.8	24	21.1	24.8	24.1	22.2
23.6	20.7	23.2	21.1	21.5	21.4	22	24.1	20.2	24.9	21.9	21.4
26.4	26.9	24.8	26.7	26.4	26.4	26	25.6	23.5	26	24.2	24.2
24.1	24.3	23.6	22.6	25.1	22.3	23	25.2	21.7	24.9	24.1	24.6
21.7	21.5	22.5	21.5	22.3	22.1	22.1	21.3	18.9	22.8	21.2	20.9
24.8	25.1	25.5	25	24.9	26.2	24.4	24.8	21.7	25.6	23.5	23.8
23.1	23	23	22.6	22.8	21.2	23.1	22.8	19.7	24.1	22.5	21.1

26.6	25.7	27.3	27.4	25.9	24.4	25.9	27.1	23	27.6	27	25.5
24.2	23.6	23.6	23.5	23.6	23.3	22.7	23.8	20.4	24.7	22.6	22.9
24.2	24.2	23.8	23.1	24.3	22.2	22.9	24.2	19.9	24.5	22.7	23.8
24.1	23.4	23	22.3	23.3	22.7	22.4	23.4	19.1	24	22	22
28.9	23	25.4	27	30.2	27.1	26.1	26.5	21	28.9	26.8	25.5
27.7	27.7	26.8	27.9	26.8	28	27.2	27.2	27.6	28.3	27.9	27.2
28.7	28.9	29.2	28.8	29.9	27.5	28.8	30	26.1	30.1	29.2	29.2
27	24.3	26	26.7	25.3	24.7	25.7	24.8	20.6	26.1	25.6	24.7
30.5	27	28.5	26.7	27.9	23.8	26.4	27.3	22.5	26.3	26.3	27.6
34.7	32.1	32.7	32.7	32.8	32.6	31.5	32.3	32.6	33.1	33.1	31.7
29.7	27.7	27	27.2	27.9	28.4	26.5	28.2	29.2	28	27.8	26.6
30.6	30.5	30.6	29.7	30.8	29.8	29.8	30.5	27.7	30.7	30.7	29.7
27.5	24.6	26.2	27.1	25.5	22.7	26.5	26.3	22.6	26.5	27.3	24.6
36.3	34.7	32	33.3	34.7	35	33.6	34.7	32	34.3	33.1	32.2
N/A	N/A	N/A	N/A	N/A	N/A	N/A	N/A	37.2	N/A	N/A	N/A
32.5	32.3	32.2	30.9	33.8	33.7	31.4	33.9	31.6	32.3	33	32
27.7	25.3	27.9	26.2	28.2	27.1	26.8	29.8	25.6	28.6	27.4	28.1
23.9	24	24	23.2	24.7	24.7	23.3	25.6	21.9	24.3	23.7	24.6
29.2	30	29.8	26.9	29.4	26.7	28.6	27.8	25.4	27	28.6	27.8
27.4	26	27.1	26.6	27.7	28.9	27.3	29	23.5	28.4	27.4	27.7
N/A	N/A	35.4	36.3	36.8	36.5	N/A	37.5	36.3	34.1	35.8	36.1
37.1	N/A	37	N/A	36.2	N/A	37.2	37	N/A	N/A	36.9	36.7
22	20.4	20.4	21.2	20.4	21.3	20.1	21.5	21.1	22	22.1	21
20.4	21.1	19.8	20.7	21.1	22.7	20.7	21.5	19.4	20.8	22.7	20.8
23.4	22.7	23.8	22.5	23.1	26.4	23	23.7	21	23.2	24.1	22.9
21.4	20.6	20.3	20.8	20.6	20.5	20.2	20.6	20.1	21.7	22.2	20.9
22	21.3	21.2	20.9	21.4	22.4	21.4	21.2	21.4	22.3	22.7	21.9
20.4	19.8	19.7	20	19.5	23.1	20.1	20.7	19.6	19	20.5	19.4
25.8	10.5	25.8	23.5	25.9	23.6	24.5	25.1	27.3	27.2	24.9	24.4
27.1	26.3	26.4	25.8	26.1	25.9	25.8	26.1	27.2	25.8	26.3	25.3
18.7	17.5	18.8	18.8	19.3	18.5	11.5	18.5	19.3	19.6	18.7	18.5
18.7	19.3	18.9	20.5	18.4	18.9	18.4	19.3	19.6	19.1	18.9	19

21.6	21.0	20.9	21.0	21.0	22.7	20.9	21.5	20.4	21.5	22.4	21.2
1.1	1.0	1.5	0.8	1.2	2.0	1.1	1.1	0.8	1.5	1.2	1.2

Table S2. Predicted gene targets of miRNAs				
hsa-miR-17-5p	hsa-miR-20a-5p	hsa-miR-20b-5p	hsa-miR-26a-5p	hsa-miR-26b-5p
CNOT6L	ZNF385A	CABLES1	USP3	USP3
TRAPPC14	ACSL4	USP3	LIF	ZNF451
NFAT5	FOXQ1	CYBRD1	ATP5F1A	GNA13
SMAD5	MAPK1	ITCH	CDK6	ZKSCAN1
BTG2	SCAMP2	SLC4A7	CDK8	PDE4B
ZBTB33	ZNF805	CDK6	WWP2	RPRD2
CABLES1	CFL2	TTPAL	ST3GAL6	REEP4
PHTF2	PPP2R2A	FRMD6	BBX	ZDHHC18
NFIB	UBR5	CLOCK	ZBTB18	DYRK1A
KCNB1	CABLES1	SHOC2	EHD1	ABHD2
DENND11	ANKFY1	MARCHF6	TBC1D13	ANKRD52
ANKRD13C	FRMD6	ARHGAP12	PDE4B	EIF5
USP3	CLOCK	RAP2C	BCL7B	KLHL24
ITCH	CSNK1A1	ACBD5	SLC38A2	SACS
FRMD6	YOD1	SESN3	CPEB4	CREBZF
SHOC2	FBXO31	LASP1	GPALPP1	ULK2
ARHGAP12	SEPTIN2	CSNK1A1	REEP4	TMCC1
RAP2C	RAB30	BBX	AGO1	FBXO28
ACBD5	DYNC1LI2	YOD1	METAP2	ICE1
SESN3	NUP35	ZBTB18	ARMH4	ZBTB20
LASP1	ENPP5	FBXO31	KPNA2	KIF21B
CSNK1A1	CHIC1	SUCO	FAXC	ZDHHC7
BBX	KLHL36	NFIB	PPP1R15B	TMEM248
FBXO31	EGLN3	RAPGEF4	MFSD14A	TBC1D13
MAP3K9	KLHL28	SEPTIN2	TP53INP1	CCSER2
RAB30	ORMDL3	CCSER2	LARP1	UBR4
PKNOX1	ARHGEF7	DYNC1LI2	PTPN13	KLHL42
VEGFA	JAK1	SLC25A44	HMGA1	SFXN1
JPT1	KLHL15	STX6	ABHD2	HECTD3
ZNF385A	DNAL1	PKNOX1	CREBRF	PRR5L
NR2C2	HOOK3	SLC22A23	UBR3	PAK1
RPA2	PHF6	KIAA0513	UBN2	SALL1
EMSY	ZBTB37	PCMTD1	TMTC3	PTP4A1
EPHA4	MASTL	CNOT6L	UBE2H	ELAVL2
TCF4	TP53INP1	LYSMD3	DST	PFKFB3
AFF1	HAUS8	VEGFA	BMP2K	TPPP
LZIC	FBXO21	TET3	MAPK6	KPNA6
AGO1	TMEM123	CLIP4	SACS	OSTM1
PHAF1	NIBAN1	PHLPP2	ARNTL2	CACNB4
KPNA2	CCND1	BTBD7	TUT4	ADAM17
BNIP2	ANKH	ATL3	ZNF608	CCND2
ORMDL3	WIPF2	ENPP5	TRPC6	SLC26A4
JAK1	NSD2	NR2C2	KLHL42	HGF
WDR82	SLC30A7	RPA2	CPEB3	USP9X
ARID4B	CREB1	EMSY	TMCC1	GSR
DNAL1	BCL2L11	USP28	RCOR1	CXADR
TMEM245	NFAT5	ARL1	GTF2A1	DAB2
KATNAL1	STAT3	QKI	CPSF2	CDV3

HOOK3	NACC2	HMGB3	TET2	PARP11
LCOR	FAM210A	ZNF202	RCBTB1	DNMBP
ZBTB37	CADM2	EPHA4	AGPAT5	GGA3
PURB	C2orf69	SLK	STRADB	MME
TP53INP1	POFUT1	TCF4	HOXA5	TDRD7
HAUS8	SIK1	EGLN3	UGGT1	KLHDC10
FBXO21	FAM117B	KLHL28	VANGL2	MED23
NIBAN1	PRR14L	CHD9	ZSWIM6	VPS13C
CDKN1A	PGM2L1	AKTIP	EPG5	PDSS2
NSD2	ZNF800	DENND5B	ZNF410	SOWAHC
RORA	AGO3	AFF1	SFXN1	TNKS
CREB1	OXR1	AGO1	CHAC1	COL12A1
KIF23	UBXN2A	TSKU	MAN2A1	SFPQ
VTI1A	TPRG1L	ZNF532	NRAS	CAMK4
ABHD2	DSTYK	RACGAP1	PIK3C2A	
MTMR3	QKI	PHAF1	PIM1	
CADM2	GAB1	RUNX3	ADAM17	
HSPA8	GID4	BNIP2	TUB	
C2orf69	TCF4	ORMDL3	MFHAS1	
SALL3	KCNB1	LPGAT1	NKX2-5	
FAM117B	TADA2B	ARHGEF7	ELAVL2	
PRR14L	ITGB8	JAK1	ETF1	
ANKRD52	RBL2	TRIM37	TXNL1	
FAM126B	CIT	EZH1	CHAF1A	
TMEM167A	WNK3	TNKS2	DCAF7	
FMNL3	ZNF652	KLHL15	SMAD1	
MIDN	KIAA0513	DDHD1	MAP1B	
AGO3	SUSD6	ATG16L1	HSPA8	
SMURF1	EFCAB14	ANKRD13C	MAP3K2	
NCOA3	IQSEC1	TRIM8	KPNA6	
UBXN2A	ANKRD12	DNAL1	RGS17	
TPRG1L	GNB5	SESN2	CEMIP2	
PRKACB	HECA	EIF4H	DYRK1A	
RLIM	LIMA1	TMEM245	EP300	
NAPEPLD	KLF3	KATNAL1	OTUD4	
FAM102A	FNBP1L	CAPRIN2	CCND2	
QKI	TSR1	BRMS1L	ESR1	
NPAS3	CENPQ	HOOK3	PTEN	
ITGB8	CMTR2	PHF6	IGF1	
CFL2	SGTB	PARD6B	CREBZF	
CIT	RAB22A	ZBTB37	ADM	
ZNF652	TNFAIP1	USP32	DDX3X	
MYO1D	ZNF70	PPP1R15B	CDC6	
IQSEC1	CEP97	MASTL	SSH2	
SACS	HMBOX1	PURB	DNMBP	
KIAA0513	PPP1R3B	FOXQ1	DNMT3B	
EFCAB14	CYBRD1	TP53INP1	SPTBN1	
FOXJ3	RAB11FIP1	ZC3H12C	ZNF772	
TNRC6B	GNS	TANC1	KLHDC10	
ANKRD12	PTPN4	HAUS8	GGA2	

ARAP2	RAB5B	MFN1	SLC25A36	
ATL3	NR2C2	FBXO21	TXLNG	
GNB5	WEE1	TMEM123	NUCKS1	
LIMA1	EEA1	NIBAN1	IRF4	
ANKFY1	SEMA7A	CCND1	BAG4	
KLF3	BCL2L2	ANKH	SFPQ	
TENT5C	EIF2S1	DNAJB6	SMAD4	
FNBP1L	MED17	CDKN1A	MYH10	
TMEM100	ARHGAP35	WAC	FKTN	
RAB22A	RABEP1	MXI1	CA2	
FHIP2A	RPS6KA5	WIPF2	RABGAP1L	
TNFAIP1	KIF23	NSD2		
ZNF70	VPS26A	RORA		
EGLN3	ENTPD4	CREB1		
SMOC1	ABL2	BCL2L11		
AKTIP	CRK	CFL2		
GID4	STX6	NFAT5		
HMBOX1	PURA	STAT3		
ARSJ	PITPNA	NACC2		
PANK3	POLR3G	SAMD8		
PPP1R3B	NCOA3	VTI1A		
KLHL36	HSPA8	PTPDC1		
PIP4K2C	SNTB2	ZNF597		
CYBRD1	HBP1	BMT2		
CHD9	MAPRE3	SPRED1		
E2F5	BAMBI	ZBTB9		
ELK4	ICMT	ABHD2		
F3	MYLIP	MTMR3		
GNS	CNOT7	HSPA8		
PTPN4	NRBP1	C2orf69		
RAB5B	NTN4	MINK1		
STAT3	RUFY2	SALL3		
MAP7	NAGK	POFUT1		
EIF2S1	SIRPA	RUNDC1		
VPS26A	SAMD12	SIK1		
ENTPD4	RGMB	PRR14L		
SCAMP2	MAP3K2	PGM2L1		
KMT2A	XIAP	ANKRD52		
DYNC1LI2	EPAS1	FAM126B		
PDLIM5	LDLR	TMEM167A		
MAP3K2	PRRG1	FMNL3		
MSMO1	TMEM64	TXLNA		
SNTB2	KATNAL1	ZNF800		
FBXO10	FBXO48	MIDN		
MAPRE3	TNRC6B	AGO3		
ICMT	C6orf120	HIF1A		
NRBP1	NABP1	OXR1		
NTN4	OTUD4	NCOA3		
RUFY2	MECP2	UBXN2A		
ELAVL2	PPP6C	TPRG1L		

OTUD4	ATXN1	NIN		
TGOLN2	MOSMO	SLC16A9		
POLR3G	SSX2IP	SEMA4B		
XIAP	SCAMP5	MKNK2		
EPAS1	SMAD7	POLQ		
EREG	DPYSL2	DSTYK		
CCND2	MAP7	FAM102A		
LDLR	FOXJ3	MAP3K3		
PPP3R1	BMPR2	GAB1		
PRRG1	MICOS10-NBL1	SAMD12		
BICD2	CDKN1A	MYLIP		
TMEM64	RLIM	HBP1		
TGFBR2	TNKS2	PHTF2		
NABP1	FMNL3	FBXO10		
KDM6B	ETV1	PEAK1		
MECP2	NUFIP2	KIF23		
ATXN1	SACS	GID4		
ZBTB4	UEVLD	SACS		
RBM20	TTPAL	TMBIM6		
FCHO2	SESN3	KMT5B		
SSX2IP	PKNOX1	ADAR		
SAMD8	CNOT6L	CEP170		
SCAMP5	VEGFA	MAPRE3		
PLEKHO2	BTBD7	GBF1		
MCL1	ATL3	KCNB1		
BCL2L2	ARL1	ACSL4		
BMPR2	HMGB3	STK17B		
MICOS10-NBL1	EPHA4	ACAP2		
WEE1	SLK	TADA2B		
RUNX3	DENND5B	FBXL5		
SLC25A44	LZIC	MKRN1		
ZNF805	KPNA2	ATAD2		
CEP97	BNIP2	CERCAM		
EZH1	LPGAT1	PAFAH1B1		
TBC1D17	EZH1	TBC1D17		
DNAJB9	PLEKHO2	KMT2B		
CLOCK	DDHD1	TMEM131L		
YOD1	ANKRD13C	ATG14		
ZBTB18	ARID4B	CIT		
FRS2	RORA	SSX2IP		
SUCO	SAMD8	ATG2A		
SEPTIN2	VTI1A	TGFBR2		
BTBD7	ABHD2	REST		
CHIC1	MTMR3	AGFG2		
ARL1	ANKRD52	ZFYVE26		
MMP2	POLQ	NETO2		
DENND5B	MFN1	WNK3		
LPGAT1	PAFAH1B1	ARID4B		
MAP3K8	TBC1D17	ARHGAP1		
KLHL15	ATG14	ZNF652		

PHF6	REST	MYO1D		
MAK16	ZFYVE26	IQSEC1		
PPP1R15B	LAPTM4A	KLHL20		
MASTL	AAK1	TNRC6A		
FOXQ1	ARAP2	FAF2		
CCND1	FEM1B	CEP57		
NACC2	CMPK1	KIAA0232		
POFUT1	DNAJC27	RB1CC1		
RUNDC1	TENT5C	PLEKHM1		
SAMD12	KIF26B	AAK1		
MYLIP	TMX3	FOXJ3		
RBL2	RSRP1	TNRC6B		
ATG14	EIF5A2	ANKRD12		
WNK3	NRIP3	ARAP2		
OGA	NIN	BICD2		
TNFRSF21	ZNFX1	UBR5		
CEP57	CRY2	RLIM		
LAPTM4A	SLC30A1	GNB5		
TTC9	SMOC1	HECA		
CMPK1	AKTIP	AKAP11		
TRIM44	PRRG4	CMPK1		
ATG2B	FYCO1	CRIM1		
CCSER2	PANK3	KLF3		
TMX3	PHAF1	DNAJC27		
SGTB	ITPKB	NAGK		
UBFD1	NPAT	ZRANB1		
FEM1C	SOX4	TENT5C		
ACVR1B	DYRK2	FNBP1L		
CRY2	E2F2	RUFY2		
CROT	CHAF1A	ATG2B		
RRAGD	ABCA1	VPS13C		
PRRG4	CAPN15	TRAPPC14		
FYCO1	GDF11	PPP6R3		
RAB11FIP1	AGO1	CMTR2		
NPAT	WDR37	ANKIB1		
PLAG1	NFIB	TMX3		
MAPK1	GIGYF1	SGTB		
DYRK2	EREG	UBFD1		
ETV1	SPOPL	FEM1C		
RND3	TMEM200C	ACVR1B		
HAS2	KDM6B	ANKRD50		
HMGB3	RACGAP1	EIF5A2		
CHAF1A	RBM20	PTGFRN		
DNAJB6	APP	NRIP3		
REST	PRKACB	NUFIP2		
GDF11	E2F3	FHIP2A		
MTF1	NHLRC3	UBC		
PITPNA	SUCO	ZNFX1		
TIMM17A	TET3	CRY2		
TRIM32	USP28	TNFAIP1		

CNOT7	TRIM8	CROT		
RGMB	CAPRIN2	HOXD11		
DPYSL2	LLPH	SLC30A1		
EIF4G2	PURB	RRAGD		
PTEN	ZC3H12C	ZNF70		
REEP3	MXI1	MCL1		
NHLRC3	TMEM167A	SMOC1		
TRIM71	CCDC71L	CRTC3		
TRIOBP	SLC16A9	PRRG4		
MOSMO	LYPD6	FYCO1		
BZW1	ZFYVE9	HMBOX1		
E2F3	AGFG2	ARSJ		
NUFIP2	PLEKHM1	PANK3		
TTPAL	ZRANB1	PPP1R3B		
STX6	FEM1C	KLHL36		
ENPP5	GPR157	PIP4K2C		
USP28	E2F5	GPR157		
SLK	DUSP2	RAB11FIP1		
TNKS2	SMAD5	E2F3		
DDHD1	MTF1	E2F5		
TRIM8	TIMM17A	ELK4		
PLXNA1	MSMO1	GNS		
LLPH	TRIM32	ITPKB		
ANKH	OSTM1	LIMK1		
DCBLD2	CCND2	M6PR		
MXI1	REEP3	MCC		
WIPF2	BICD2	NPAT		
FAM210A	C14orf28	PDGFB		
BMT2	ANKRD33B	PLAGL2		
PGM2L1	BZW1	PLS1		
SLC16A9	ZBTB9	PPP6C		
POLQ	CERCAM	MAPK1		
DSTYK	KLHL20	PTPN4		
APP	FOXJ2	RAB5B		
GAB1	ACVR1B	SKI		
MFN1	CEP120	GPR137B		
PAFAH1B1	PLS1	WEE1		
TMEM131L	TGFBR2	ULK1		
AGFG2	LRP12	EEA1		
WAC	TANC1	DYRK2		
ZFYVE26	TXLNA	SEMA7A		
YES1	ITCH	MAP7		
UBE3C	TSKU	BCL2L2		
PLEKHM1	SLC4A7	E2F2		
AAK1	MARCHF6	EFNB2		
ZRANB1	ZBTB18	EIF2S1		
NRIP3	AFF1	MED17		
NIN	ZNF532	DUSP2		
ZNFX1	PLXNA1	EPHB4		
PPP6C	USP32	ARHGAP35		

SOX4	DNAJB6	FOXK2		
E2F2	BMT2	RABEP1		
SOCS6	MINK1	RPS6KA5		
DUSP2	FAM126B	VPS26A		
ABCA1	HIF1A	ENTPD4		
ZBTB6	SEMA4B	MECP2		
WDR37	MKNK2	E2F1		
OSTM1	MAP3K3	HAS2		
TET3	PEAK1	CHAF1A		
PKD2	ADAR	ABCA1		
CTSA	CEP170	CAPN15		
FBXO48	GBF1	TOPORS		
TMEM200C	KMT2B	MTF1		
ANKRD33B	ATG2A	PITPNA		
TBC1D2	PHLPP2	TIMM17A		
TMBIM6	PPP6R3	POLR3G		
CERCAM	MCL1	MAP3K2		
ZBTB7A	F3	ZBTB6		
CLIP4	SKI	MSMO1		
SMAD6	MAP3K5	SNTB2		
HBP1	MLLT1	BTG2		
RACGAP1	ZBTB6	ZBTB33		
RNF145	ZBTB33	FICD		
ZNF532	FBXL5	TRIM32		
SESN2	ATAD2	DNAJB9		
CAPRIN2	PKD1	BAMBI		
ZBTB9	RB1	ICMT		
ZC3H12C	EIF4G2	CNOT7		
LRP12	TMEM131L	NRBP1		
RUNX1	TBC1D15	LRP12		
OXR1	ANKRD50	WDR37		
CEP170	TNFRSF21	OSTM1		
TSKU	KMT5B	ESR1		
SLC4A7	MAGOHB	KDM6B		
UBR5	FRS2	ELAVL2		
ACSL4	BBX	ATXN1		
TANC1	RPA2	OTUD4		
RPS6KA5	TMEM245	RGMB		
MAP3K3	RBL1	TGOLN2		
MAP3K12	RUNX1	GIGYF1		
ADAR	KIAA0232	XIAP		
GBF1	CRIM1	DPYSL2		
TADA2B	ARSJ	EIF4G2		
FBXL5	PDGFB	EREG		
ATAD2	PLAGL2	OCRL		
KMT2B	FOXK2	PTEN		
TNRC6A	RAPGEF4	RB1		
SIPA1L3	FICD	LDLR		
AKAP11	OCRL	PPP3R1		
NAGK	DNAJC28	REEP3		

VPS13C	RB1CC1	SPOPL		
CMTR2	GPR137B	TMEM64		
PTGFRN	ATG16L1	ACER2		
SLC30A1	SESN2	NHLRC3		
TBC1D15	WAC	C14orf28		
ITPKB	MAP3K12	ZNF805		
LIMK1	STK17B	FBXO48		
PLAGL2	MKRN1	C6orf120		
PLS1	NETO2	NABP1		
SKI	TNRC6A	TRIOBP		
KAT2B	AKAP11	DNAJC28		
ARHGAP35	CHD9	TMEM200C		
TSG101	UNK	U2SURP		
RAPGEF4	CAV1	UNK		
OCRL	SKIL	ZBTB4		
STK11	DNM1L	RBM20		
KLF10	LIAS	SLC30A7		
KIAA0232	CEP43	ANKRD33B		
ANKRD50	ZNF107	MOSMO		
HIF1A	TMEM9B	F3		
MFN2	ZDHHC20	BMP2		
TXLNA	KIAA1191	BMPR2		
PCMTD1	PTPRO	MICOS10-NBL1		
PHLPP2	PPP1R12B	BZW1		
TRIM37	GINS4	UXS1		
MINK1	FAXC	PKMYT1		
SEMA4B	NFATC2IP	TFAM		
NETO2	RCCD1	DNM1L		
MKNK2	ZNF280B	LIAS		
KMT5B	SGMS1	CEP43		
HOXD11	DLC1	SSH2		
M6PR	PSD3	ZNF107		
PDGFB	PLEKHM3	TMEM9B		
RBL1	VPS53	ADAT2		
FOXK2	VEZF1	ZDHHC20		
FICD	C9orf40	KIAA1191		
DNAJC28	RBM41	CDIN1		
GPR137B	ENTPD7	MAPK9		
SLC22A23	MAVS	TXNIP		
KLHL28	TMEM267	GRAMD1A		
ATG16L1	NAA50	PPP1R12B		
BRMS1L	F2R	RPF2		
USP32	LRPAP1	GINS4		
STK17B	SRSF2	FAXC		
MKRN1	TFAM	NFATC2IP		
UNK	ZNF264	RCCD1		
CPOX	F2RL3	ZNF280B		
F2RL3	PNPLA4	YTHDC1		
TFAM	NBL1	NIPA1		
DNM1L	BTN3A2	SGMS1		

ADAT2	CBX5	XIRP2		
KIAA1191	CDIN1	MFSD8		
ZNF417	YTHDC1	GABPB1		
FAS	CNOT4	BTN3A1		
PTPRO	AP1G1	RBM12B		
PPP1R12B	MUC17	PSD3		
RCCD1	CCP110	MAP3K14		
YTHDC1	CERS2	VPS53		
MAPK9	MAPK9	CAMTA1		
SGMS1	MFSD8	CCP110		
PSD3	SH3GLB1	SH3GLB1		
MAP3K14	GABPB1	ERAP1		
VPS53	SCD	TMEM138		
CAMTA1	ASB1	PDZD11		
ZNF107	CAMTA1	C9orf40		
GABPB1	C18orf32	GBP3		
POGK	GPAM	RBM41		
C9orf40	PBXIP1	TMEM242		
RBM41	BTN3A1	ENTPD7		
TMEM242	RBM12B	MAVS		
ENTPD7	ADGRL3	SERF1A		
TMEM9B	SERF1A	SERF1B		
MAVS	SERF1B	ZFYVE21		
EPB41L5	GLO1	VCPKMT		
ZFYVE21	RFK	SIKE1		
SIKE1	CBX1	NAA50		
F2R	CSDE1	EIF4A2		
LRPAP1	EIF4A2	F2R		
SRSF2	CKAP2	LRPAP1		
UGCG	ERAP1	SRSF2		
VDAC1	VDAC1	ZNF264		
PNPLA4	SMAD4	F2RL3		
CEP43	NIPA1	B2M		
COA1	RPF2	COIL		
CDIN1	TXNIP	PNPLA4		
CNOT4	PDZD11	KCNJ8		
AP1G1		SCD		
PUDP		SMAD4		
BTN3A2		SKIL		
CCP110		GLO1		
SH3GLB1		CBX1		
LIAS		SLC5A3		
FER		BTN3A2		
TXNIP		CSDE1		
GINS4		CBX5		
FAXC		CNOT4		
MFSD8		ACADSB		
VCPKMT		ASB1		
NAA50		TMEM267		
ZNF264		BMP8B		

SLC5A3		CPOX		
BTN3A1		AP1G1		
CBX5		MUC17		
TMEM267		SMIM13		
LPIN1		GPAM		
PBXIP1				
NFATC2IP				
RBM12B				
ADARB1				
SERF1A				
SERF1B				
GLO1				
CSDE1				
SSH2				
FAM8A1				
ERAP1				
TMEM138				
EPS15L1				
EIF4A2				
COIL				
DCTN6				
KRT10				
CLEC12B				
SCD				
XIRP2				
ASB1				
RPF2				
ZNF280B				
B2M				

hsa-miR-193b-3p	hsa-miR-205-5p	hsa-miR-223-3p	hsa-miR-328-3p	hsa-miR-485-5p
ANKFY1	ETF1	LIF	STT3A	MEF2D
E2F6	RAN	PHF19	SMC1A	WDTC1
EBAG9	MAP3K9	PTBP2	ADNP	S100A16
ABI2	BCL6	SPPL2A	TMEM132B	CDKN1A
ARMC1	ETNK1	IL6ST	AGO1	ZNF641
BAZ2A	LPCAT1	NFIA	ARL6IP1	CSNK2A1
CLSTN1	ITGA5	SMARCD1	HIF1AN	MIDEAS
MSANTD2	PTPRJ	WASL	H2AX	SSH3
SMIM14	VEGFA	E2F1	MMP16	QSOX1
ZNF618	DDX5	LMO2	RPS9	ZNF81
IL17RD	B4GALT6	FBXW7	GTPBP2	BAZ2A
WDR82	B4GALT5	FOXO1	CDC42EP1	NT5DC3
ATOH8	TM9SF2	SCARB1	TAPBP	ATOH8
ARHGAP19	LCOR	PRDM1	ORMDL1	PLCD3
KRAS	EZR	TRPV2	PLCE1	TSTD2
MYLK	LYN	NLRP3	PTPRJ	TEX261
CCND1	CCNJ	SECISBP2L	SFRP1	SUMF2
YWHAZ	ZEB2	TP53	LPCAT3	HIF3A
GXYLT1	LYSMD3	STMN1	IVNS1ABP	PPARGC1A
MCL1	HMGB1	SEPTIN2		CDC42BPA
MAP3K3	PARD6B	RIF1		FOSL2
STMN1	CREB1	TWF1		DVL3
SELENON	SATB2			TMPRSS4
TSC1	YES1			MAP7D1
TGFBR3	RAB11FIP1			DDN
GDF11	RAP2B			APOA5
RSF1	PRKCE			TRAPPC2
TNFRSF21	IPO7			PGPEP1
ZBTB5	MMD			IKZF3
BICD2	PTEN			LPIN1
CTDSPL2	PICALM			PSD
ALKBH5	RUNX2			MDM4
INO80D	LRRK2			KIAA0513
TMEM30A	AFF4			SNX27
NUFIP2	LAMC1			HOOK3
LAMC1	E2F1			PLEKHG4B
CDK17	SMAD1			MOB3A
PLAU	PHC2			APOBEC3F
PPP2R5C	LRP1			ATP6V0D2
STX16	XPR1			MFSD4A
SNRPD3	ZEB1			CLEC17A
ETS1	PTPRM			TFCP2L1
PLXNC1	ACSL1			HEYL
DCAF7	VPS52			PACS2
NSF	CENPF			TMEM19
DYRK2	ERBB3			KIAA1328
FAF1	AMOT			CCL22
CNOT6	RTN3			ARHGDIA

KIT	ENPP4			DFFA
PTEN	NOTCH2			TFDP2
IGFBP5	MDH2			CRIPT
NT5DC3	MGLL			HMGA2
ADCY9	DMXL2			FBXW2
LRRC8A	SERINC3			GOLGA3
MAPK8	SMAD2			ARSA
MDH2	SQLE			CASTOR2
ELMO2	STK38L			ZBTB8OS
NQO2	AFF1			ZNF587
DCTN5	SHISA6			CGNL1
PSRC1	LMNA			RFT1
ZMAT3	TCF20			DTX3L
CKAP2L	CFAP65			MMS22L
TXLNA	CLIP1			KIAA0408
NF2	RBBP4			CDKAL1
AREL1	E2F5			CD209
ADARB1	NIPA2			LHPP
CCDC28A	HSPA8			NDRG3
HHAT	BDP1			SOAT1
STARD7				NAV1
RGMA				PLEKHA1
PLEKHA2				SHISA2
PTPN9				SLC25A53
MED21				CD300LG
SHMT2				TMEM170A
AIMP2				ANGPT4
NAGA				CLCC1
ZNF71				SLC38A7
RPL27A				URM1
TMPPE				SLC24A4
SLC30A7				SLC27A1
				PLCXD1
				NRIP3
				PHACTR4
				IRGQ
				SHOX
				ZNF805
				ZNF500
				RBM4B
				EGF
				ITCH
				PLEKHA6
				CLDN12
				TMBIM6
				ZBTB8A
				USP22
				KIF6
				ARMH3
				SLX4IP

hsa-miR-489-3p	hsa-miR-181d-5p
GNAL	OSBPL3
PAX3	PHC3
PROX1	CPEB4
USP46	CPEB4
SOX4	ZNF121
FNIP1	CHCHD7
FAM117B	CHCHD7
WDR26	CPEB4
CADM2	OSBPL3
NUFIP2	LONRF1
SPIN1	ZNF268
KLHL28	ZNF699
SLC7A11	FSD1L
CITED2	FSD1L
FEM1B	RAN
POU2F1	TNRC6B
UBE2D1	INO80D
PPIL4	INO80D
IRS2	ARSJ
	KPNA1
	TFRC
	LMAN1
	ZFP36L2
	FSD1L
	FKBP1A
	TNRC6B
	MIDEAS
	MIDEAS
	ADCY9
	FSD1L
	TNRC6B
	ZNF268
	ZNF268
	ZNF268
	ZNF268
	ZNF268
	SSX2IP
	RPS6KA3
	RPS6KA3
	RPS6KA3
	RPS6KA3
	RPS6KA3
	RPS6KA3
	GSKIP
	GSKIP
	FSD1L
	FSD1L
	RAN

	SLC10A7
	PHC3
	CPEB4
	CPEB4
	CPEB4
	SLC10A7
	CHCHD7
	CPEB4
	ZNF268
	ZNF268
	RLIM
	MIDEAS
	DRAM1
	TBL1XR1
	FSD1L
	FSD1L
	FSD1L
	FSD1L
	ZNF844
	TBL1XR1
	RLIM
	GSKIP
	KRBOX4
	DRAM1
	ZBTB4
	TBL1XR1
	PHC3
	PHC3
	TMEM94
	TBL1XR1
	FSD1L
	FSD1L
	TBL1XR1
	IL1A
	ZNF121
	SLC10A7
	KRBOX4
	FSD1L
	FSD1L
	ZNF268
	ZNF268
	ZNF268
	ZNF268
	ZNF268
	ZNF268
	TBL1XR1
	GSKIP
	SLC10A7
	ZADH2
	ZADH2

	SLC10A7
	KLHL15
	PURB
	ZNF440
	ZNF268
	TNPO1
	C2orf69
	ZADH2
	DDX3X
	ZNF844
	DDX3X
	TMEM94
	RAP1B
	SLC25A37
	DDIT4
	KPNA1
	TNPO1
	DUSP5
	ZFP36L2
	ETS1
	DDX3X
	DDX3X
	TNPO1
	TNPO1
	ID4
	ARF6
	RAP1B
	TNRC6B
	SLC10A7
	HEPHL1
	ZBTB4
	KRBOX4
	ZNF844
	ETS1
	ETS1
	ZNF268
	ZNF268
	ZNF268
	ZNF268
	ZNF268
	FKBP1A
	RAP1B
	RAP1B
	RAP1B
	RAP1B
	ZFAND6
	TUBB
	SLC10A7
	SLC10A7
	SLC25A25

	ATM
	RPS6KA3
	TBL1XR1
	RCOR1
	ATM
	ATM
	ATM
	ATP2B1
	ATM
	ATP2B1
	TUBB
	TUBB
	RAP1B
	PALS1
	ATP2B1
	RAP1B
	RAP1B
	RAP1B
	RAP1B
	LCLAT1
	RAP1B
	RNF6
	TUBB
	TNRC6B
	TNRC6B
	DYNC1LI2
	EMSY
	CPEB4
	TFRC
	DCBLD2
	ZNF597
	TNPO1
	LCLAT1
	ATM
	CHMP2B
	DDX3X
	DDX3X
	HECW2
	HECW2
	WASHC5
	SPIRE1
	HECW2
	FNDC3B
	FNDC3B
	FNDC3B
	ARSJ
	GOLGA1
	GOLGA1
	SLC7A1
	ZNF268

	ETS1
	ETS1
	LMAN1
	DYNC1LI2
	ZNF266
	TMF1
	G3BP2
	G3BP2
	RLF
	WASHC5
	HECW2
	HECW2
	DDX3X
	DDX3X
	ATP2B1
	ID4
	GRK2
	ATP2B1
	LCLAT1
	KDM5A
	CCNK
	SPIRE1
	SPIRE1
	ZBTB4
	CCNQ
	PTBP3
	ZNF268
	TMEM94
	TMEM94
	TMEM94
	TMEM94
	ZFAND6
	ZFAND6
	ZFAND6
	ZFAND6
	ZFAND6
	ZNF266
	ZNF83
	CPEB4
	CPEB4
	CPEB4
	PTPDC1
	C2orf69
	CARM1
	PRRC2C
	TMEM94
	TMEM94
	TMEM94
	ZNF266
	FNDC3B

	PRRC2C
	ZNF83
	HECW2
	ZBTB4
	PER2
	TNPO1
	ZNF268
	SCAMP2
	DYNC1LI2
	ETS1
	TMF1
	TNPO1
	TNPO1
	CARM1
	ZNF266
	ZNF266
	ZNF266
	PRRC2C
	CCDC88C
	ZNF83
	TFRC
	ATXN7
	FNDC3B
	ETS1
	ETS1
	ZNF268
	ZNF268
	ATXN7
	ZFAND6
	ZFAND6
	PPP2R5E
	PPP2R5E
	OSBPL3
	OSBPL3
	OSBPL3
	PRRC2C
	HECW2
	PRRC2C
	OSBPL3
	HECW2
	ZNF268
	RPS6KA3
	PPP2R5E
	SLC19A2
	TMEM94
	HECW2
	ATXN7
	ATXN7
	PRRC2C
	ID4

	ATXN7
	GOLGA8B
	CCDC88C
	SLC35G2
	ZNF268

Table S3: Univariable survival analysis for identified miRNAs using median expression as a cut-off point.

miRNA		All	HR (univariable)
miR-17-5p	High	9 (56.2)	0.76 (0.08-7.37, p=0.817)
	Low	7 (43.8)	
miR-193b-3p	High	8 (50.0)	0.25 (0.03-2.45, p=0.236)
	Low	8 (50.0)	
miR-205-5p	High	8 (50.0)	1.01 (0.14-7.17, p=0.994)
	Low	8 (50.0)	
miR-20a-5p	High	8 (50.0)	2.11 (0.29-15.13, p=0.456)
	Low	8 (50.0)	
miR-20b-5p	High	8 (50.0)	0.52 (0.05-4.99, p=0.569)
	Low	8 (50.0)	
miR-223—3p	High	10 (62.5)	0.36 (0.04-3.48, p=0.379)
	Low	6 (37.5)	
miR-26a-5p	High	8 (50.0)	0.25 (0.03-2.45, p=0.236)
	Low	8 (50.0)	
miR-26b-5p	High	9 (56.2)	0.36 (0.04-3.48, p=0.379)
	Low	7 (43.8)	
miR-328-3p	High	9 (56.2)	1.09 (0.15-7.74, p=0.933)
	Low	7 (43.8)	
miR-485-5p	High	8 (50.0)	0.85 (0.12-6.02, p=0.867)
	Low	8 (50.0)	
miR-489-3p	High	8 (50.0)	0.00 (0.00-Inf, p=0.999)
	Low	8 (50.0)	

Supplementary file. Correlation between the expression of AR and selected miRNAs in TNBCs

1. TCGA Data:

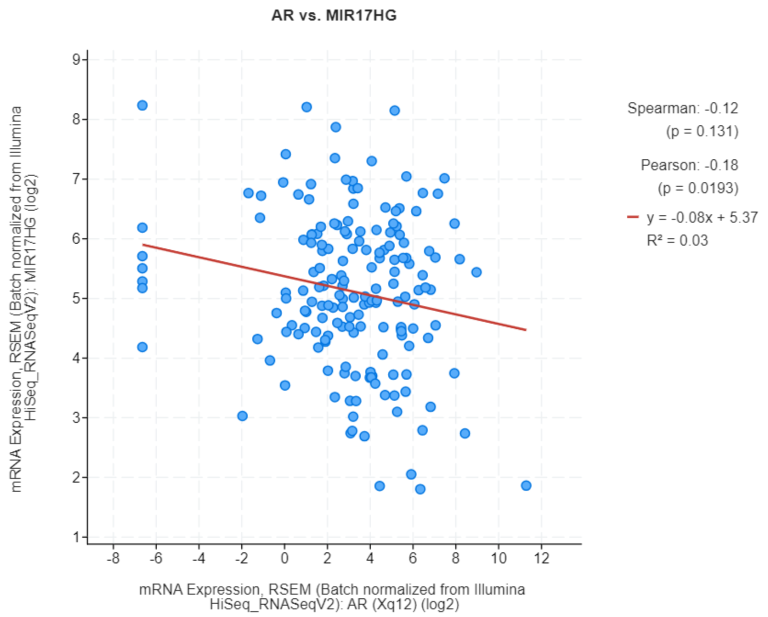

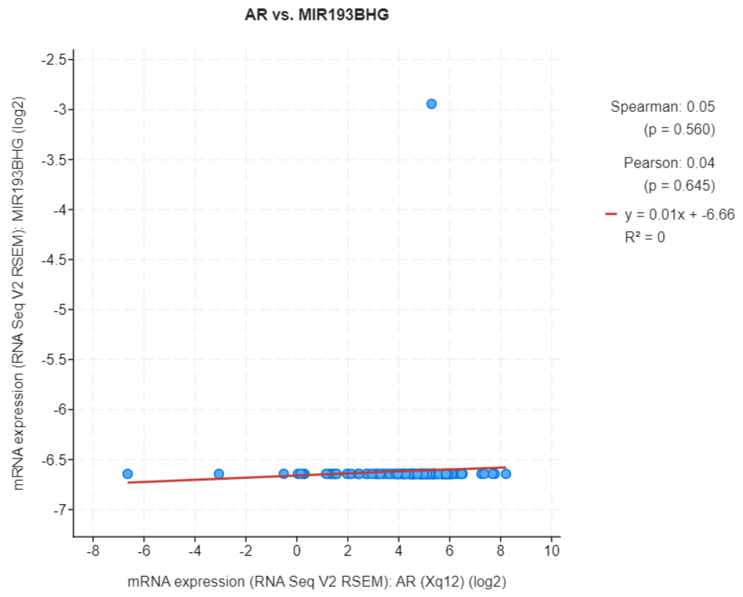

2. GSE19783 Data:

Source: https://www.ncbi.nlm.nih.gov/geo/query/acc.cgi?acc=GSE19783

Correlation Matrix

		AR	hsa-miR-17	hsa-miR-193b	hsa-miR-20a	hsa-miR-20b	hsa-miR-328	hsa-miR-485-5p	hsa-miR-489	hsa-miR-223	hsa-miR-26a	hsa-miR-26b	hsa-miR-181d
AR	Spearman's rho	—											
	p-value	—											
hsa-miR-17	Spearman's rho	-0.121	—										
	p-value	0.667	—										
hsa-miR-193b	Spearman's rho	0.039	-0.118	—									
	p-value	0.893	0.676	—									
hsa-miR-20a	Spearman's rho	-0.089	0.946	0.004	—								
	p-value	0.753	< .001	0.995	—								
hsa-miR-20b	Spearman's rho	-0.05	0.954	0.021	0.989	—							
	p-value	0.863	< .001	0.944	< .001	—							
hsa-miR-328	Spearman's rho	0.421	-0.332	0.218	-0.25	-0.218	—						
	p-value	0.119	0.226	0.434	0.368	0.434	—						
hsa-miR-485-5p	Spearman's rho	-0.025	0.007	0.114	-0.05	-0.057	0.225	—					
	p-value	0.934	0.985	0.686	0.863	0.842	0.419	—					
hsa-miR-489	Spearman's rho	0.296	-0.325	0.129	-0.332	-0.35	0.371	0.689	—				
	p-value	0.283	0.237	0.648	0.226	0.201	0.173	0.006	—				
hsa-miR-223	Spearman's rho	0.271	0.479	0.382	0.593	0.579	-0.029	-0.114	-0.064	—			
	p-value	0.327	0.073	0.161	0.022	0.026	0.923	0.686	0.822	—			
hsa-miR-26a	Spearman's rho	0.261	0.443	-0.079	0.439	0.457	0.107	-0.211	-0.346	0.257	—		
	p-value	0.347	0.1	0.783	0.103	0.089	0.705	0.45	0.206	0.354	—		
hsa-miR-26b	Spearman's rho	0.279	0.536	0.082	0.625	0.614	0.25	-0.111	-0.05	0.579	0.414	—	
	p-value	0.314	0.042	0.773	0.015	0.017	0.368	0.695	0.863	0.026	0.126	—	
hsa-miR-181d	Spearman's rho	-0.368	0.132	0.075	0.182	0.161	-0.089	0.086	0.196	-0.014	-0.05	-0.061	—
	p-value	0.178	0.639	0.793	0.515	0.567	0.753	0.763	0.482	0.964	0.863	0.832	—

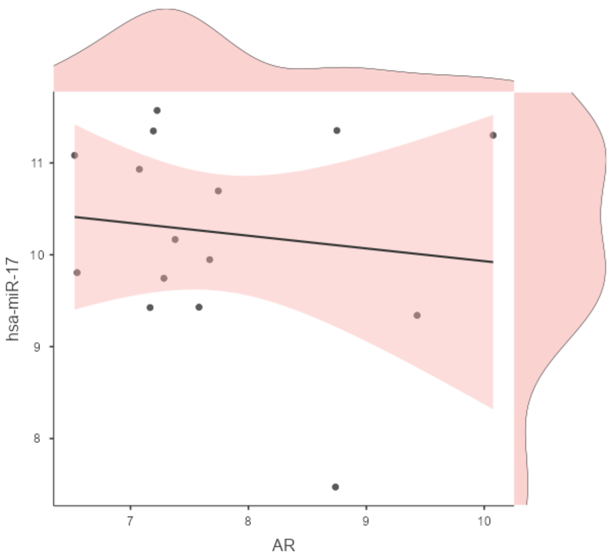

hsa-miR-17
11
10
9
8
7
8
9
10
AR

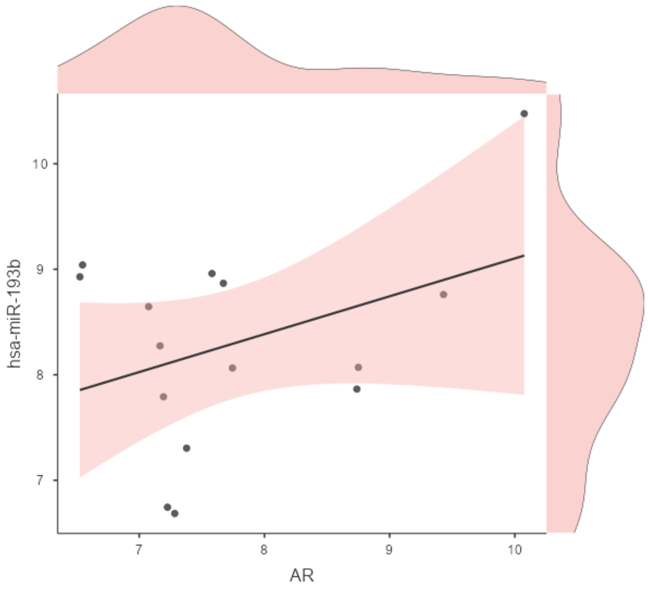

hsa-miR-193b
10
9
8
7
7
8
9
10
AR

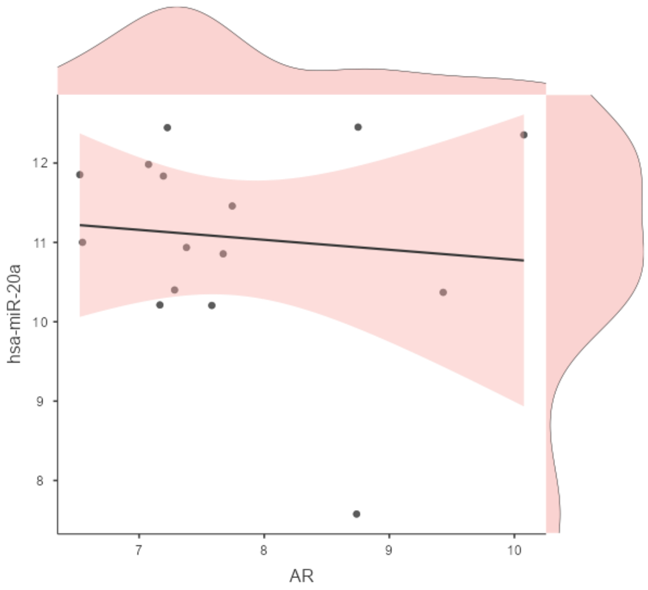

hsa-miR-20a
12
11
10
9
8
7
8
9
10
AR

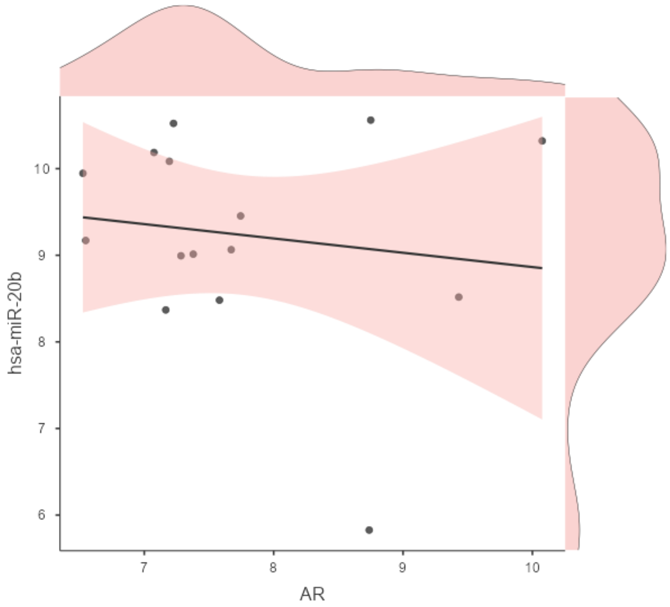

hsa-miR-20b
10
9
8
7
6
7
8
9
10
AR

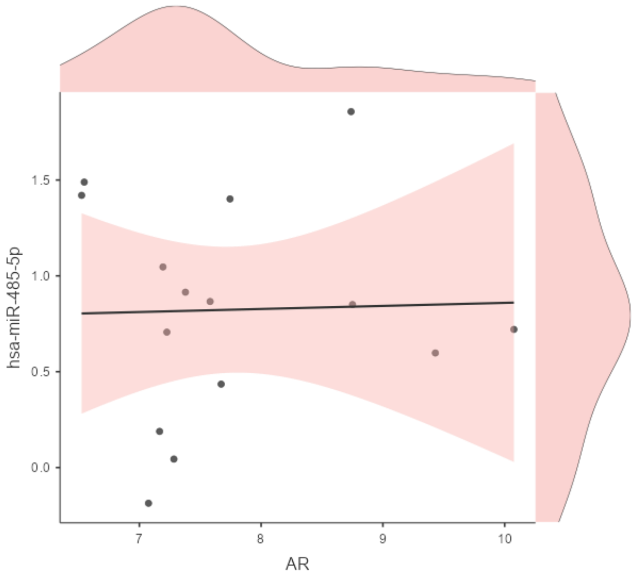

hsa-miR-485-5p
1.5
1.0
0.5
0.0
7
8
9
10
AR

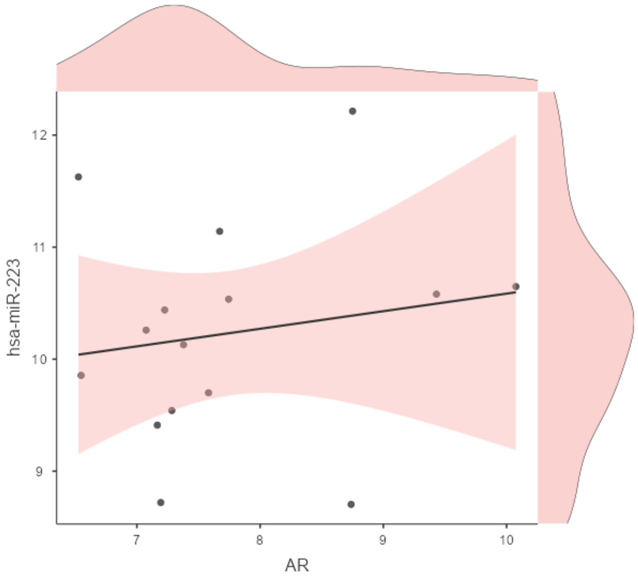

hsa-miR-223
12
11
10
9
7
8
9
10
AR

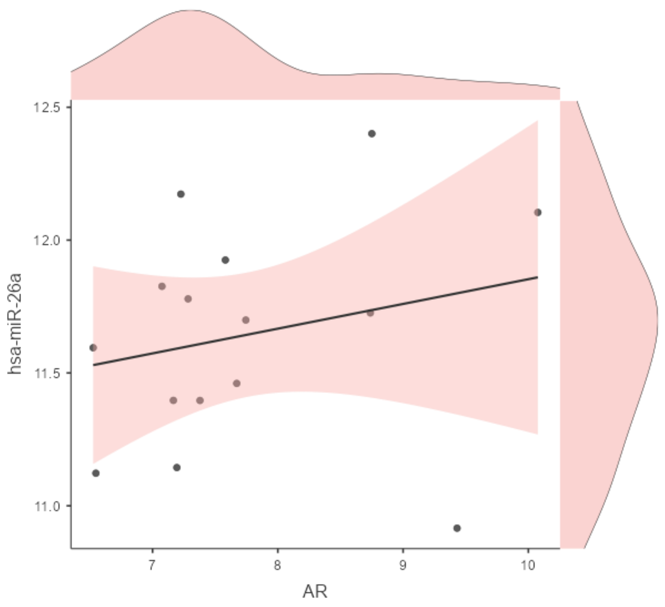
12.5
12.0
11.5
11.0
hsa-miR-26a
7
8
9
10
AR

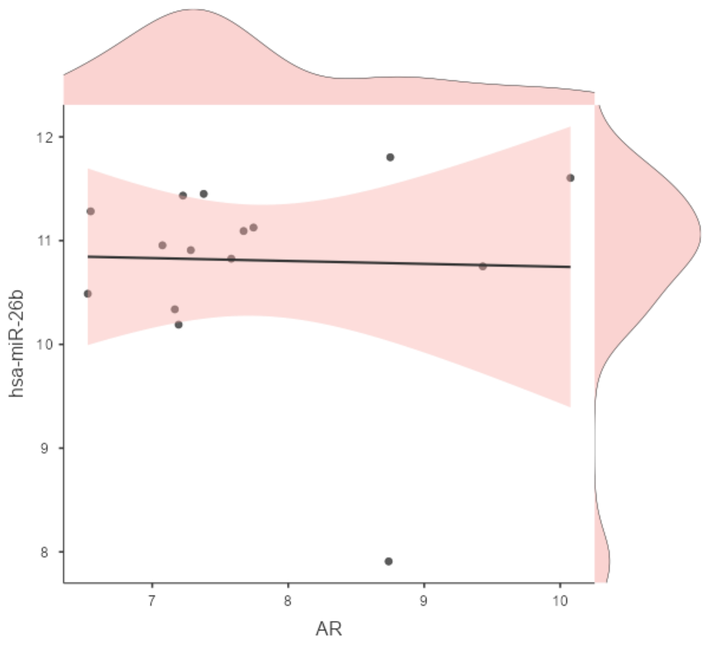
12
11
10
9
8
hsa-miR-26b
7
8
9
10
AR

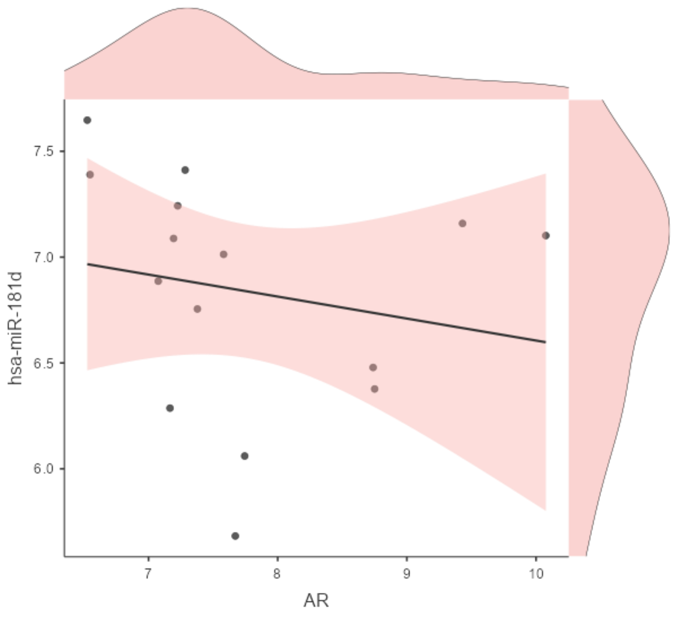
7.5
7.0
6.5
6.0
hsa-miR-181d
7
8
9
10
AR

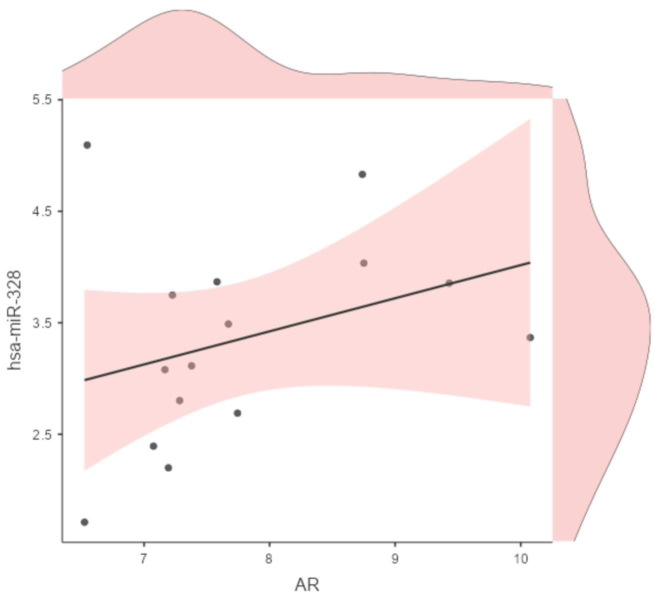

hsa-miR-328
5.5
4.5
3.5
2.5
7
8
9
10
AR

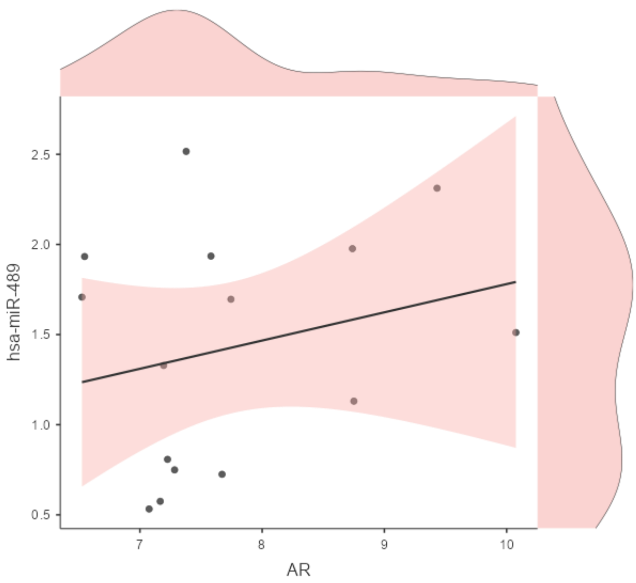

hsa-miR-489
2.5
2.0
1.5
1.0
0.5
7
8
9
10
AR

Clustering analysis for AR and selected miRNAs:

- Mir-328, mir-489, mir-485, and mir-193b cluster together with higher expression in the AR high group – in accordance with our data

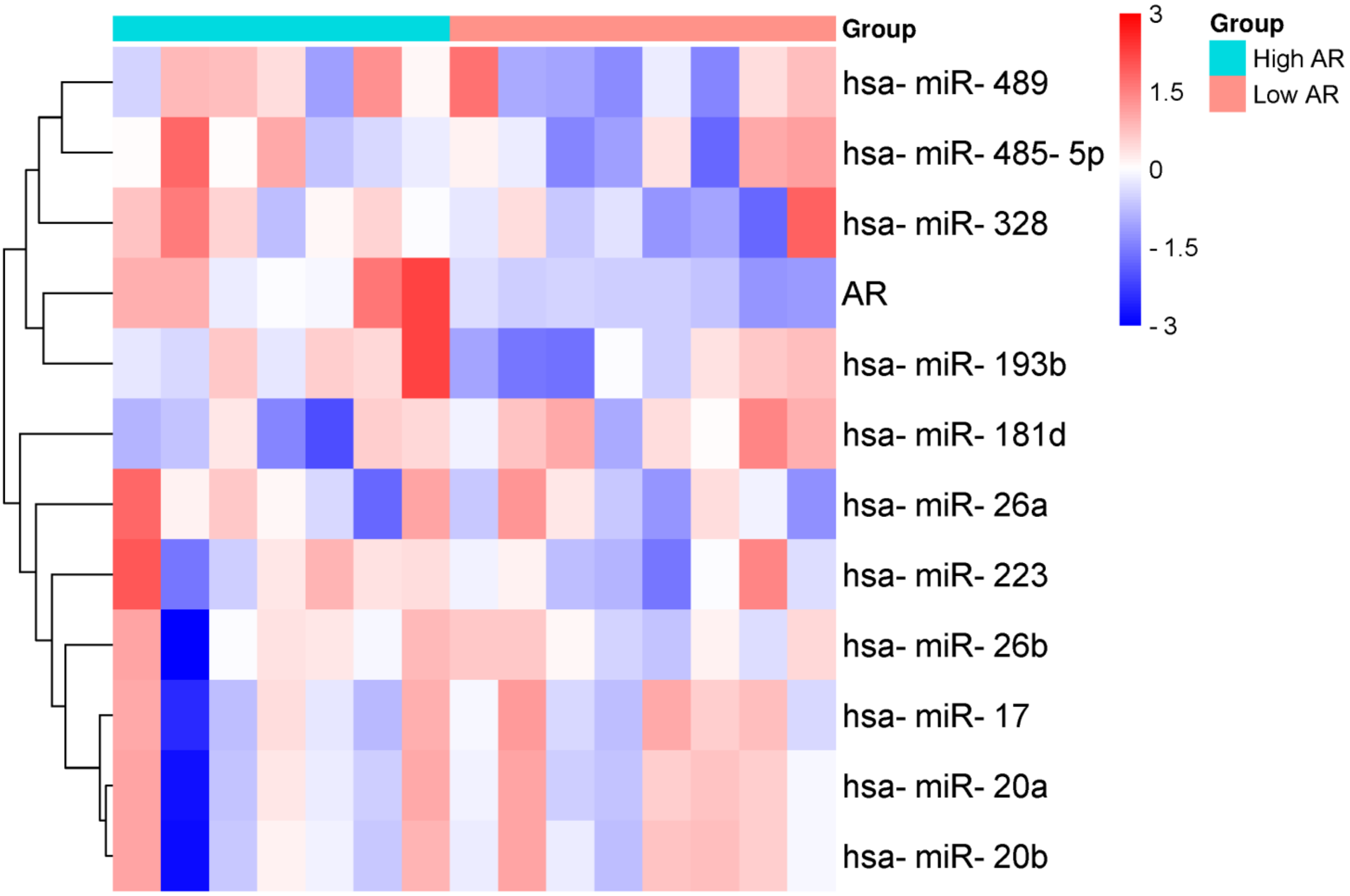

Supplement: Supplementary file 1 [file cancers-16-00665-s001.zip › cancers-2793622-supplementary.pdf]
